# Postpartum obesity and weight gain among human immunodeficiency virus‐infected and human immunodeficiency virus‐uninfected women in South Africa

**DOI:** 10.1111/mcn.12949

**Published:** 2020-01-13

**Authors:** Angela M. Bengtson, Tamsin K. Phillips, Stanzi M. le Roux, Kirsty Brittain, Allison Buba, Elaine J. Abrams, Landon Myer

**Affiliations:** ^1^ Department of Epidemiology Brown University School of Public Health Rhode Island; ^2^ Division of Epidemiology and Biostatistics, School of Public Health and Family Medicine University of Cape Town Cape Town South Africa; ^3^ ICAP, Mailman School of Public Health and Department of Pediatrics, Vagelos College of Physicians & Surgeons Columbia University New York City New York USA

**Keywords:** anthropometry, HIV and nutrition, HIV, maternal obesity, maternal postpartum weight loss, pregnancy

## Abstract

In South Africa, up to 40% of pregnant women are living with human immunodeficiency virus (HIV), and 30–45% are obese. However, little is known about the dual burden of HIV and obesity in the postpartum period. In a cohort of HIV‐uninfected and HIV‐infected pregnant women initiating antiretroviral therapy in Cape Town, South Africa, we examined maternal anthropometry (weight and body mass index [BMI]) from 6 weeks through 12 months postpartum. Using multinomial logistic regression, we estimated associations between baseline sociodemographic, clinical, behavioural, and HIV factors and being overweight–obese I (BMI 25 to <35), or obese II‐III (BMI >35), compared with being underweight or normal weight (BMI <25), at 12 months postpartum. Among 877 women, we estimated that 43% of HIV‐infected women and 51% of HIV‐uninfected women were obese I‐III at enrollment into antenatal care, and 51% of women were obese I‐III by 12 months postpartum. On average, both HIV‐infected and HIV‐uninfected women gained, rather than lost, weight between 6 weeks and 12 months postpartum, but HIV‐uninfected women gained more weight (3.3 kg vs. 1.7 kg). Women who were obese I‐III pre‐pregnancy were more likely to gain weight postpartum. In multivariable analyses, HIV‐infection status, being married/cohabitating, higher gravidity, and high blood pressure were independently associated with being obese II‐III at 12 months postpartum. Obesity during pregnancy is a growing public health concern in low‐ and middle‐income countries, including South Africa. Additional research to understand how obesity and HIV infection affect maternal and child health outcomes is urgently needed.

**Key messages**

- In a cohort of HIV‐uninfected and HIV‐infected pregnant women initiating antiretroviral treatment obesity was common; 47% were obese (BMI >30) at their first antenatal care visit, and 51% were obese at 12 months postpartum.
- On average, both HIV‐infected and HIV‐uninfected women gained weight between 6 weeks and 12 months postpartum, but weight gain was lower among HIV‐infected women. Postpartum weight gain was most common among women who were obese at their first antenatal care visit.
- Women who were married/cohabiting, had higher gravidity, or high blood pressure at enrollment in antenatal care were *more* likely to be obese II or III (BMI ≥35), whereas HIV‐infected women were *less* likely to be obese II or III at 12 months postpartum.

## 1 INTRODUCTION

Globally, many low‐ and middle‐income countries are facing increasingly complex burdens of disease, with the rise of noncommunicable conditions alongside ongoing burdens of infectious diseases. For example, in South Africa, the prevalence of human immunodeficiency virus (HIV) is remarkably high, whereas obesity and related conditions are major public health concerns (Karim et al., 2011; Kharsany et al., 2015; Sartorius, Veerman, Manyema, Chola, & Hofman, 2015). Pregnancy is a critical time when both obesity and HIV can impact women and infants' health during the perinatal period and beyond (Aviram, Hod, & Yogev, 2011; Begum, Sachchithanantham, & De Somsubhra, 2011; Brocklehurst & French, 1998; Malaba et al., 2017; Marchi, Berg, Dencker, Olander, & Begley, 2015; Xiao et al., 2015). Postpartum weight retention, although heterogeneous in nature, has the potential to contribute significantly to the obesity epidemic in South Africa (Chetty, Carter, Bland, & Newell, 2014). In some areas of South Africa, up to 40% of pregnant women are living with HIV, and 30–45% of pregnant women are obese (Basu, Jeketera, & Basu, 2010; Davies et al., 2012; Kharsany et al., 2015; Stephanie V. Wrottesley, Ong, Pisa, & Norris, 2018). Despite the importance of comanaging both obesity and HIV during pregnancy and the postpartum period to optimize maternal and child health outcomes, little research has examined postpartum weight change in routine care settings with a high burden of HIV (Anderson et al., 2018; Basu et al., 2010; Cames et al., 2014).

Obesity during pregnancy is a well‐known risk factor for complications during pregnancy, adverse pregnancy outcomes, an influences ongoing maternal and child health (Aviram et al., 2011; Begum et al., 2011; Marchi et al., 2015). Obese women may be at higher risk for gestational weight gain during pregnancy, subsequent postpartum weight retention, and the development of hypertension and diabetes in women (Begum et al., 2011; Rong et al., 2015). Until recently, for women living with HIV, undernutrition rather than obesity has been the primary concern during pregnancy (Grinspoon et al., 1998; Karim et al., 2011; Kindra, Coutsoudis, & Esposito, 2011; Lartey, 2008; Villamor et al., 2006; Widen et al., 2013; Widen et al., 2019; Young et al., 2012). However, with rapid urbanization, changing diets, and improved access to lifelong combination antiretroviral therapy (ART), the nutritional status of HIV‐infected women during pregnancy may be changing and postpartum weight retention becoming more common (Chetty et al., 2014; Meintjes et al., 2015; Meintjes et al., 2017; Murnane et al., 2010; Villamor et al., 2006; Wilkinson et al., 2015; S. V. Wrottesley, Pisa, & Norris, 2017). In order to develop effective health promotion approaches in countries experiencing dual epidemics of HIV and obesity, a clear understanding of factors that influence postpartum body mass index (BMI) and weight retention among pregnant HIV‐infected and HIV‐uninfected women is needed.

The aim of this paper is to describe maternal anthropometry, including weight, BMI, and weight change from 6 weeks through 1 year postpartum in a cohort of HIV‐infected women on ART and HIV‐uninfected women in South Africa. Additionally, we explore sociodemographic, clinical, HIV, and behavioural factors associated with being overweight or obese at 12 months postpartum and postpartum weight change.

## 2 METHODS

### 2.1 Study setting and population

To address this aim, we conducted a secondary analysis using data from HIV‐infected women enrolled in the Strategies to Optimize ART Services for maternal and child health (MCH‐ART) trial and a parallel cohort of HIV‐uninfected pregnant women (HIV‐unexposed–uninfected study) conducted in Cape Town, South Africa. Details of both studies have been published previously (le Roux et al., 2019; Myer et al., 2016; Myer et al., 2018). Briefly, the two cohorts had similar inclusion and exclusion criteria, enrolling HIV‐infected pregnant women initiating ART and HIV‐uninfected pregnant women who were > 18 years of age between March 2013 and August 2015 at their first antenatal care (ANC) visit at a primary care center in Gugulethu in Cape Town. Women who were breastfeeding at their first postpartum visit, scheduled 7 days after delivery, were enrolled and followed through 12 months postpartum. Out of 1,087 mother–infant pairs screened at their first postpartum visit, 92 women (79 HIV‐infected women and 14 HIV‐uninfected women; 8% overall) were excluded due to not breastfeeding (le Roux et al., 2019).

Gugulethu is an urban community of approximately 300,000 people outside of Cape Town and is characterized by high levels of poverty and HIV (Myer et al., 2018; Strategic Development Information & GIS Department, 2013). Over 95% of women in this setting receive ANC prior to delivery (Myer et al., 2015). Provision of ART and prevention of mother‐to‐child transmission (PMTCT) services are provided at no cost as a part of routine ANC at all public sector clinics, in accordance with local guidelines. Starting in 2013, all HIV‐infected women attending ANC were eligible for lifelong ART, regardless of CD4 count of WHO clinical stage (WHO, 2013). All HIV‐infected women in the study initiated the local first‐line ART regimen of tenofovir (300 mg) + emtricitabine (200 mg)/lamivudine (300 mg) + efavirenz (600 mg), provided as a fixed‐dose combination pill taken once daily.

HIV‐uninfected and HIV‐infected women initiating ART were included in the present analysis if they met eligibility criteria, were followed through 12 months postpartum (*n* = 884), and had a singleton pregnancy (*n* = 7 excluded). Participants completed study visits at enrollment into ANC (baseline), delivery, 6 weeks, and 3, 6, 9, and 12 months postpartum.

### 2.2 Measures

Maternal anthropometry was assessed by maternal weight (in kilograms), BMI (calculated as kilograms/meters^2^), and changes in maternal weight from 6 weeks (baseline) through 12 months postpartum. Postpartum weight change was defined as either no weight change (within +/−2 kg), weight loss more than 2 kg, or weight gain more than 2 kg, between 6 weeks postpartum and each time point. Women were weighed, and their height was measured at enrollment into ANC (median gestational age 20 weeks, range 4–39) and at all postpartum visits by trained data collectors following standard operating procedures. For example, women were weighed on a calibrated digital scale (such as the Charder MS7301 250 Kg Digital Scale) with their shoes and extra layers of clothing removed and were measured using a portable stadiometer (Seca 213 Stature Meter Free‐Standing Stand). Competency checks, repeat training, and random quality control checks were conducted throughout the study period. BMI was categorized as underweight (<18.5), normal (18.5 to <25), overweight (25 to <30), obese I (30 to <35), obese II (35 to <40), and obese III (>40). Maternal weight at delivery was not measured; therefore, we were not able to estimate gestational weight gain. In addition, information on the development of gestational diabetes, pregnancy‐induced hypertension, preeclampsia, or postpartum hypertension or diabetes was not available.

At enrollment into ANC, information on baseline sociodemographic, clinical, behavioural, and HIV disease (if applicable) characteristics was collected. Gestational age at enrollment into ANC was determined using ultrasound. A composite poverty score developed by our research team, calculated from current employment, housing type and access to household assets, was used to categorize women as “most,” “moderate,” or “least” disadvantaged (Brittain et al., 2017). Perinatal depression was measured using the Edinburgh Postnatal Depression Scale (range 0–30; Chorwe‐Sungani & Chipps, 2017; Cox, Holden, & Sagovsky, 1987). A score of >13 was used to indicate probable depression (Redinger, Norris, Pearson, Richter, & Rochat, 2018). Alcohol use was measured using the 3‐item Alcohol Use Disorders Identification Test‐Consumption (AUDIT‐C; range 0–12). For women, an AUDIT‐C score >3 indicates hazardous drinking (Bush, Kivlahan, McDonell, Fihn, & Bradley, 1998). Blood pressure was measured at baseline and categorized as normal (<120/80 mm Hg), elevated (systolic 120–129 and diastolic <80 mm Hg), Stage 1 hypertension (systolic 130–139 or diastolic 80–89 mm Hg), or Stage 2 hypertension (systolic >140 or diastolic >90 mm Hg; American College of Obstetricians and Gynecologists, 2018). Due to the few women who had Stage 2 hypertension, women with Stages 1 and 2 hypertension were combined in statistical analyses. Among HIV‐infected women, CD4 cell count (<200, 201 to <350, 350 to <500, >500 cells/mm^3^) and viral load (<1,000, 1,000 to <10,000, and >10,000 copies/ml), timing of HIV diagnosis (during the current pregnancy or previously) and use of antiretroviral prophylaxis for PMTCT in a previous pregnancy, and previous combination ART use were assessed at enrollment into ANC.

### 2.3 Statistical analyses

The goals of the statistical analysis were to describe maternal weight, BMI, and weight change overtime and by HIV status during the postpartum period, as well as to estimate associations between demographic, clinical, behavioural factors and HIV status, and being overweight or obese at 12 months postpartum. Maternal anthropometry overtime and by HIV status was examined descriptively and graphically. To explore associations with being overweight or obese, we categorized women into one of three groups: underweight or normal weight (BMI <25), overweight or obese I (BMI 25 to <35), or obese II or III (BMI >35). Because only 4% of the population was underweight, effect estimates could not be estimated separately for this group. Therefore, we grouped underweight women with normal weight women in order to retain as much of the sample as possible to maximize statistical precision. We used multinomial logistic regression to estimate odds ratios (OR) for associations between baseline factors and being overweight–obese I (outcome 1), or obese II‐III (outcome 2), compared with being underweight or normal weight (referent), at 12 months postpartum.

In bivariable analyses, all factors with a *p* value <.05 were considered for inclusion into the multivariable model. All variables considered for inclusion into the multivariable model were evaluated for collinearity using pairwise correlation coefficients. For variables with a pairwise correlation coefficient >.50, we selected the variable with the stronger bivariable association for inclusion into the multivariable model. When evaluating associations with BMI at 12 months postpartum, BMI at first ANC visit was highly collinear with BMI at 12 months postpartum and, therefore, was not included due to model convergence issues. Gestational age at enrollment into ANC and breastfeeding duration are likely intermediary variables, between several variables in the model and BMI category at 12 months postpartum, and therefore were not included in models (Ananth & Schisterman, 2017; Hernandez‐Diaz, Schisterman, & Hernan, 2006).

As an exploratory, secondary analysis, we explored predictors of change in maternal weight (categorized as weight loss, no weight change, and weight gain using the category definition above) between 6 weeks postpartum and 12 months among the 596 women with a weight measurement at 6 weeks and 12 months postpartum. Bivariable and multivariable analyses were analogous to those described above. Among HIV‐infected women (*n* = 464), we examined whether HIV‐related factors, including timing of HIV diagnosis, previous PMTCT during pregnancy, viral load, and CD4 count, were associated with BMI at 12 months postpartum. Previous ART use was not included due to the majority of participants initiating ART for the first time.

Missing BMI and blood pressure data at enrollment into ANC were common (10–12%) and more frequent among HIV‐infected women. To address potential bias due to missing data, we conducted a sensitivity analysis where we used multiple imputation to impute all missing data from a multivariate normal distribution (*N* = 50 imputations; Rubin, 1987). We then explored associations between baseline factors and BMI at 12 months postpartum in the imputed data. All statistical analyses were conducted in Stata version 15 (StataCorp, College Station, TX).

### 2.4 Ethical approval

Ethical approval for both the MCH‐ART and HU2 studies was provided by the University of Cape Town's Human Research Ethics Committee. The MCH‐ART study also received ethical approval from the Columbia University Institutional Review Board.

## 3 RESULTS

**Figure 1:**
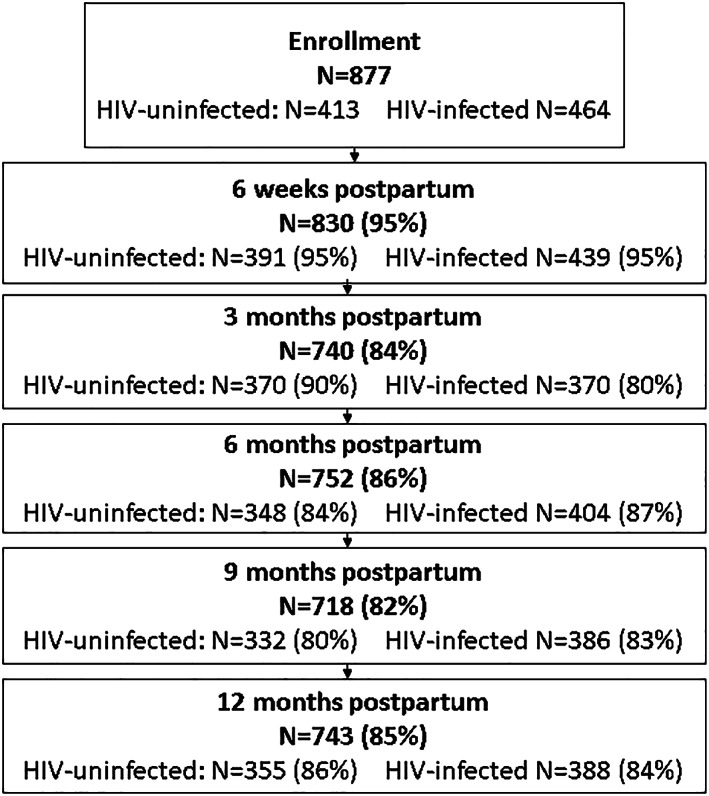
Study population of human immunodeficiency virus (HIV)‐infected and HIV‐uninfected women from enrollment at first antenatal care visit through 12 months postpartum

**Figure 2:**
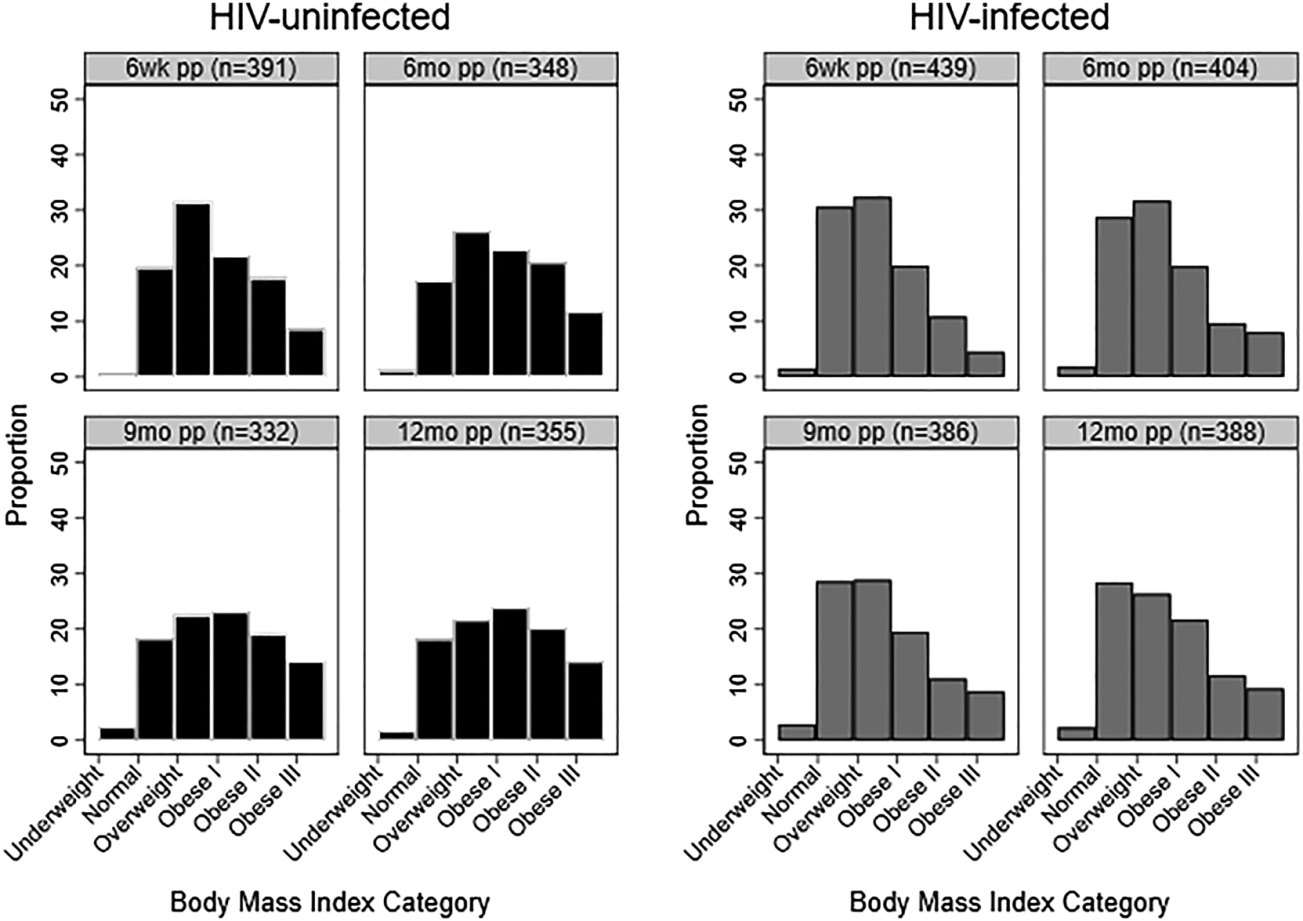
Body mass index (BMI) category by human immunodeficiency virus (HIV) status and study visit, 6 weeks through 12 months (mo) postpartum (pp). BMI categories are underweight (<18.5 kg/m^2^), normal (18.5 to <25), overweight (25 to <30), obese I (30 to <35), obese II (35 to <40), and obese III (>40)

We included 877 HIV‐uninfected (*n* = 413, 47%) and HIV‐infected women initiating ART (*n* = 464, 53%) who were breastfeeding 7 days after delivery (Figure 1). At enrollment into ANC, 47% of women were obese I‐III (Table 1). By 12 months postpartum, 51% of women were obese I‐III, including 43% of HIV‐infected women (Figure 2). The mean age at enrollment was 28 years (*SD* 5.8), and the mean gestational age was 20 weeks (*SD* 7.8, range 4–39 weeks). HIV‐infected women were less likely to be employed (39% among HIV‐infected women, compared with 47% among HIV‐uninfected), to have a secondary education (24 vs. 45%) and to be obese I‐III pre‐pregnancy (36 vs. 45%), but were more likely to have Stage 1 or 2 hypertension (33 vs. 22%) and report hazardous alcohol use (25 vs. 7%). Among HIV‐infected women, the vast majority (96%) were initiating ART for the first time, and nearly half had a viral load >10,000 copies/ml (48.7%) and a CD4 cell count <350 cells/mm^3^ (49.6%) at enrollment into ANC.

**Table 1:** Characteristics at enrollment into antenatal care among 877 pregnant women receiving maternity care services in Cape Town, South Africa

	Total	HIV uninfected	HIV infected	*p* value
Characteristic	*N* = 877	*N* = 413	*N* = 464	
**Maternal anthropometry**				
	Median (*SD*)			
Height, cm	158.6 (6.5)	158.6 (6.5)	158.5 (6.6)	.90
Weight, kg	76.4 (17.5)	78.1 (18.1)	74.7 (16.6)	<.01
BMI, kg/m^2^	30.4 (6.7)	31.0 (6.9)	29.7 (6.5)	<.01
	*N* (%)			
BMI category				.15
Underweight (<18.5)	10 (1.3)	5 (1.3)	5 (1.3)	
Normal (18.5 to <25.0)	163 (20.5)	73 (18.3)	90 (22.8)	
Overweight (25.0 to <30.0)	247 (31.1)	116 (29.1)	131 (33.2)	
Obese I (30.0 to <35.0)	185 (23.3)	97 (24.3)	88 (22.3)	
Obese II (35.0 to <40.0)	117 (14.8)	64 (16.0)	53 (13.5)	
Obese III (≥40.0)	71 (9.0)	44 (11.0)	27 (6.9)	
				
**Demographic and Clinical Characteristics**				
	Mean (*SD*)			
Maternal age	28.3 (5.8)	28.1 (6.1)	28.5 (5.5)	.29
Gravidity	1.9 (1.2)	2.4 (1.2)	1.4 (1.1)	<.01
Gestational age, completed weeks	20 (7.8)	21 (7.6)	20 (7.9)	.06
	*N* (%)			
Education				<.01
Less than secondary	580 (66.1)	228 (55.2)	352 (75.9)	
Secondary or higher	297 (33.9)	185 (44.8)	112 (24.1)	
Employment				.01
Unemployed	501 (57.1)	218 (52.8)	283 (61.0)	
Employed	376 (42.9)	195 (47.2)	181 (39.0)	
Poverty category				.02
Most disadvantaged	284 (32.4)	121 (29.3)	163 (35.1)	
Moderate disadvantage	332 (37.8)	177 (42.9)	155 (33.4)	
Least disadvantaged	261 (29.8)	115 (28.8)	146 (31.5)	
Planned current pregnancy				.10
No	606 (69.1)	274 (66.3)	332 (71.5)	
Yes	271 (30.9)	139 (33.7)	132 (28.5)	
Marital status				.31
Not married/cohabitating	502 (57.2)	229 (55.5)	273 (58.8)	
Married/cohabitating	375 (42.8)	184 (44.5)	191 (41.2)	
Primigravida				.03
No	688 (78.5)	311 (75.3)	377 (81.3)	
Yes	189 (21.5)	102 (24.7)	87 (18.7)	
Perinatal depression^a^				.12
No probable depression	800 (91.4)	384 (93.0)	416 (90.0)	
Probable depression	75 (8.6)	29 (7.0)	46 (10.0)	
Alcohol use^b^				<.01
Below threshold	729 (83.3)	383 (92.7)	346 (74.9)	
Hazardous drinking	146 (16.7)	30 (7.3)	116 (25.1)	
Blood pressure category^c^				<.01
Normal	394 (51.2)	213 (56.8)	181 (45.8)	
Elevated	163 (21.2)	78 (20.8)	85 (21.5)	
Stage 1 or 2 high	213 (27.7)	84 (22.4)	129 (32.7)	
**HIV characteristics (N = 464)**					
	N (%)			
HIV diagnosis				
Before this pregnancy	—	—	197 (42.5)	NA
During this pregnancy	—	—	267 (57.5)	NA
PMTCT prophylaxis in a previous pregnancy				
No	—	—	264 (70.0)	NA
Yes	—	—	113 (30.0)	NA
Previous combination ART				
No	—	—	446 (96.1)	NA
Yes	—	—	18 (3.9)	NA
Viral load, copies/ml				
<1,000			74 (16.0)	NA
1,000 to <10,000			164 (35.3)	NA
≥ 10,000			226 (48.7)	NA
CD4 count, cells/mm^3^				
≤200	—	—	74 (16.4)	NA
201 to ≤350	—	—	150 (33.2)	NA
351 to ≤500	—	—	110 (24.3)	NA
≥500	—	—	118 (26.1)	NA

*Note*. *p* values compare HIV uninfected and HIV infected and are calculated using chi‐square test for categorical variables and *t*‐tests for continuous variables.

a Based on the Edinburgh Postnatal Depression Scale (range 0–30); a score of ≥13 indicates probable depression.

b Based on the AUDIT‐C (range 0–12); a score of ≥3 indicates hazardous drinking.

c Blood pressure categorized as: normal (<120/80 mm Hg), elevated (systolic 120–129 and diastolic <80 mm Hg), or Stage 1 high (systolic 130–139 or diastolic 80–89 mm Hg) or Stage 2 hypertension (systolic >140 or diastolic >90 mm Hg). Missing data: height *n* = 16 (1.8%), weight at enrollment *n* = 82 (9.4%), BMI at enrollment *n =* 84 (9.4%), gestational age at enrollment *n* = 4 (0.5%), perinatal depression *n* = 2 (0.2%), alcohol use *n* = 2 (0.2%), blood pressure *n* = 107 (12.2%), previous antiretroviral prophylaxis *n* = 87 (18.8%), CD4 cell count *n* = 12 (2.6%).

Abbreviations: ART, antiretroviral therapy; BMI, body mass index; HIV, human immunodeficiency virus; NA, not applicable; PMTCT, prevention of mother‐to‐child HIV transmission.

### 3.1 Maternal anthropometry

Compared with HIV‐infected women, HIV‐uninfected women weighed more and had a higher median BMI (median weight 75 vs. 70 kg and median BMI 29.7 vs. 27.8 kg/m^2^) at 6 weeks postpartum and at 12 months postpartum (median weight 79 vs. 72 kg and median BMI 31.6 vs. 28.6 kg/m^2^; Table 2). Although HIV‐infected women were lighter overall, the median BMI among HIV‐infected women at all time‐points from 6 weeks postpartum through 12 months postpartum was overweight (BMI 25 to <30) (Table 2, Figure 2).

**Table 2:** Maternal anthropometry including (A) weight, (B) body mass index, and (C) maternal weight change overtime, by HIV status

A.	Maternal weight (kg)–Median (IQR)		
	6 weeks postpartum	6 months postpartum	12 months postpartum
HIV uninfected	75.4 (65.6, 89.1)	78.5 (66.1, 93.0)	78.6 (66.6, 94.5)
HIV infected	70.0 (60.2, 81.6)	70.1 (60.7, 82.3)	72.1 (60.1, 84.5)

B.	Maternal BMI (kg/m^2^)–Median (IQR)		
	6 weeks postpartum	6 months postpartum	12 months postpartum
HIV uninfected	29.7 (25.9, 35.2)	31.1 (26.6, 36.8)	31.6 (26.8, 37.3)
HIV infected	27.8 (24.1, 32.2)	27.8 (24.1, 32.6)	28.6 (24.3, 33.4)

C.	Maternal weight change (kg)^a^, from 6 weeks postpartum–Median (IQR)		
	6 months postpartum	9 months postpartum	12 months postpartum
HIV uninfected	2.2 (−0.6, 5.6)	3.1 (−1.0, 7.2)	3.3 (−0.9, 8.2)
HIV infected	0.6 (−2.0, 3.3)	1.1 (−2.0, 5.7)	1.7 (−2.2, 7.4)

a Negative value indicates weight loss from 6 weeks postpartum; positive value indicates weight gain from 6 weeks postpartum.

Abbreviations: HIV, human immunodeficiency virus; IQR, interquartile range.

**Figure 3:**
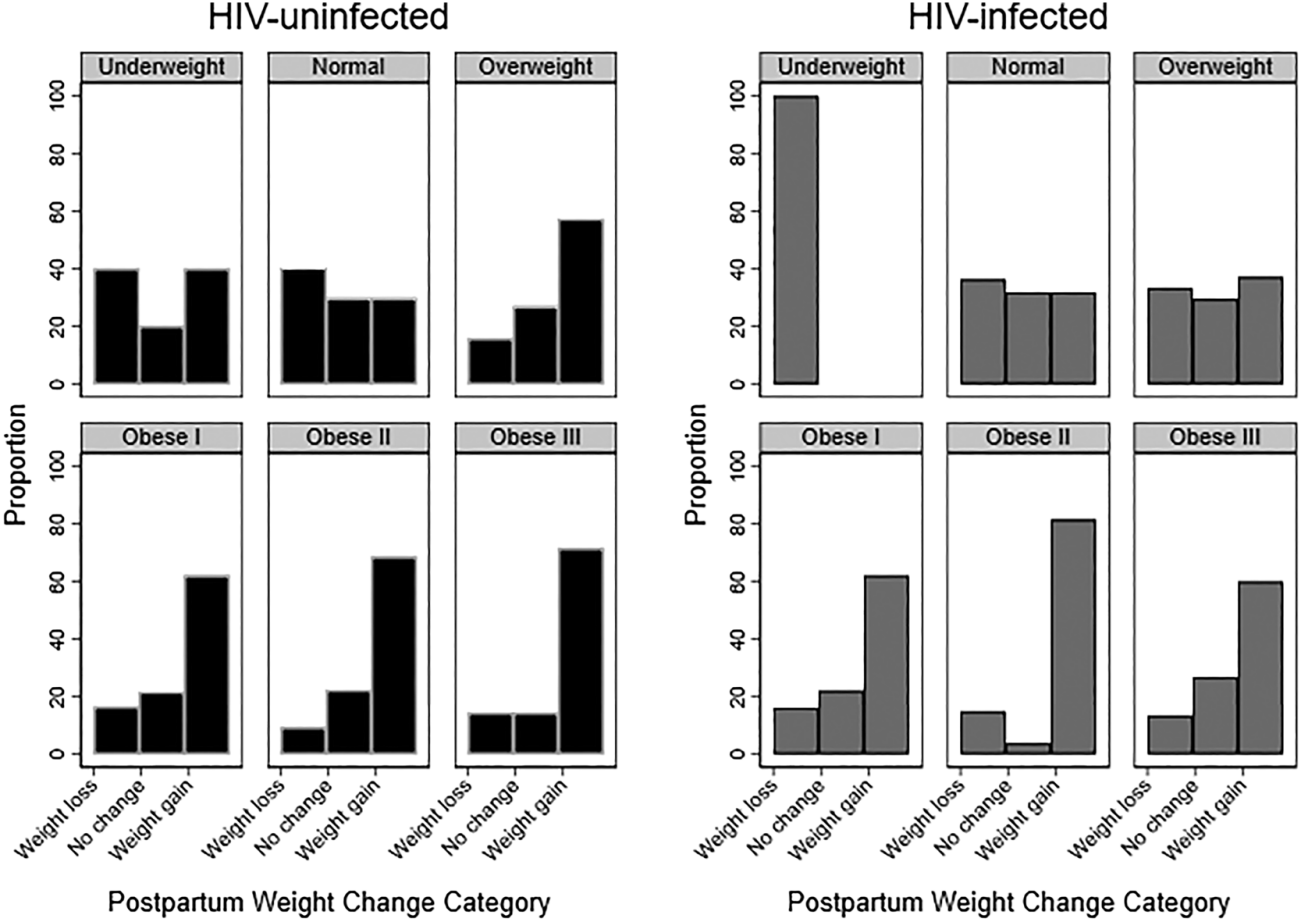
Postpartum weight change from 6 weeks through 12 months postpartum by body mass index (BMI) category at first antenatal care visit and human immunodeficiency virus (HIV) status. BMI categories are underweight (<18.5 kg/m^2^), normal (18.5 to <25), overweight (25 to <30), obese I (30 to <35), obese II (35 to <40), and obese III (>40). Weight change between 6 weeks postpartum and 12 months postpartum were defined as no weight change (within +/−2 kg of weight at 6 weeks postpartum), weight loss more than 2 kg, or weight gain more than 2 kg

On average, both HIV‐infected and HIV‐uninfected women gained weight between 6 weeks postpartum and 12 months postpartum. However, HIV‐infected women gained less weight during the postpartum period. For example, between 6 weeks and 12 months postpartum, HIV‐uninfected women gained a median of 3.3 kg (IQR −0.9, 8.2), whereas HIV‐infected women gained a median of 1.7 kg (IQR −2.2, 7.4). Postpartum weight gain varied by pre‐pregnancy BMI category, with women who were obese I‐III prior to pregnancy being more likely to gain weight between 6 weeks and 12 months postpartum (Figure 3).

### 3.2 Associations with BMI at 12 months postpartum

In multivariable analyses, women with Stage 1 or 2 hypertension at enrollment into ANC were more likely to be overweight–obese I at 12 months postpartum (Table 3). Women with elevated or Stage 1 or 2 hypertension, higher gravidity, and who were married or cohabitating were more likely to be obese II‐III at 12 months postpartum. HIV‐infected women initiating ART were less likely to be obese II‐III at 12 months postpartum, compared with HIV‐uninfected women. Effect estimates were similar in sensitivity analyses using multiple imputation to account for missing data (Table S1).

**Table 3:** Bivariable and multivariable associations for the relationship between characteristics at enrollment into antenatal care and being overweight/obese I or obese II/III, compared with underweight or normal (referent) at 12 months postpartum among HIV‐infected and HIV‐uninfected women in South Africa

Characteristics at enrollment into antenatal care	Overweight or obese I (BMI 25 to < 35), compared with underweight or normal (BMI <25)		Obese II or III (BMI ≥35), compared with underweight or normal (BMI <25)	
	Bivariable OR (95% CI)	Multivariable OR (95% CI)	Bivariable OR (95% CI)	Multivariable OR (95% CI)
HIV status				
HIV uninfected	1.00	1.00	1.00	1.00
HIV infected	0.68 (0.47, 0.99)*	0.73(0.47, 1.14)	0.40 (0.26, 0.60)*	0.42 (0.25, 0.71)*
Education				
Less than secondary	1.00	—	1.00	—
Secondary or higher	0.92 (0.62, 1.36)	—	1.07 (0.69, 1.64)	—
Employment				
Unemployed	1.00	1.00	1.00	1.00
Employed	1.48 (1.01, 2.16)*	1.37 (0.90, 2.09)	1.43 (0.94, 2.17)	1.36 (0.84, 2.21)
Poverty category				
Most disadvantaged	1.00	—	1.00	—
Moderate disadvantage	0.74 (0.48, 1.15)	—	1.03 (0.62, 1.70)	—
Least disadvantaged	0.74 (0.46, 1.18)	—	1.17 (0.69, 1.98)	—
Planned current pregnancy				
No	1.00	—	1.00	—
Yes	1.15 (0.77, 1.72)	—	1.52 (0.98, 2.36)	—
Marital status				
Not married/cohabitating	1.00	1.00	1.00	1.00
Married/cohabitating	1.44 (0.98, 2.11)	1.39 (0.90, 2.16)	2.55 (1.67, 3.90)*	2.37 (1.45, 3.90)*
Maternal age	1.05 (1.02, 1.09)*	—	1.09(1.05, 1.14)*	—
Gravidity, continuous	1.33 (1.13, 1.57)*	1.19 (0.98, 1.45)	1.60 (1.34, 1.92)*	1.29 (1.04, 1.61)*
Perinatal depression				
No probable depression	1.00	—	1.00	—
Probable depression	0.72 (0.39, 1.34)	—	0.50 (0.23, 1.07)	—
AUDIT‐C categories				
Below threshold	1.00	—	1.00	—
Hazardous drinking	1.02 (0.63, 1.65)	—	0.79 (0.45, 1.38)	—
Blood pressure category				
Normal	1.00	1.00	1.00	1.00
Elevated	1.61 (0.95, 2.72)	1.67 (0.98, 2.83)	2.76 (1.55, 4.93)*	2.96 (1.62, 5.41)*
Stage 1 or 2 high	2.30 (1.34, 3.39)*	2.46 (1.42, 4.29)*	4.79 (2.69, 8.53)*	5.90 (3.20, 10.90)*

*Note*. Multivariable associations are adjusted for all other covariates listed in the table.

Abbreviations: AUDIT, alcohol use disorders identification test; BMI, body mass index; CI, confidence interval; HIV, human immunodeficiency virus; OR, odds ratio.

* *p* < .05.

Findings were somewhat similar when examining factors associated with postpartum weight change between 6 weeks postpartum and 12 months postpartum, but fewer predictors were identified. Only being primigravida (OR 2.69, 95% CI 1.38, 5.25) and reporting hazardous alcohol use (OR 3.22, 95% CI 1.53, 6.77) were associated with weight loss, whereas Stage 1 or 2 hypertension (OR 1.97, 95% CI 1.17, 3.31) was associated with weight gain between 6 weeks and 12 months postpartum (Table S2).

Among HIV‐infected women, we found no evidence of associations between HIV‐specific factors, including baseline CD4 count, viral load, timing of HIV diagnosis, previous PMTCT use, and being overweight–obese I or obese II‐III at 12 months postpartum (Table S3).

## 4 DISCUSSION

In this urban South African population of HIV‐uninfected and HIV‐infected pregnant women initiating ART, 47% were obese I‐III at their first ANC visit and 51% by 12 months postpartum. Compared with HIV‐uninfected women, HIV‐infected women initiating ART had a lower BMI pre‐pregnancy, which persisted through 12 months postpartum. However, the median BMI among HIV‐infected women was overweight throughout the follow‐up period. Conversely, HIV‐infected women were more likely to have hypertension at baseline, compared with HIV‐uninfected women. On average, both HIV‐infected and HIV‐uninfected women gained, rather than lost, weight through 12 months postpartum, but HIV‐infected women gained less weight. Postpartum weight gain was also more common among women who were obese at their first ANC visit. In multivariable analyses, HIV infection, being married/cohabiting, higher gravidity, and elevated blood pressure at enrollment were associated with being either obese II or II, compared with normal weight or underweight, at 12 months postpartum.

In high‐resource settings, the rising prevalence of obesity throughout pregnancy and postpartum weight retention are well‐recognized risk factors for poor maternal and child health outcomes and increased healthcare service utilization (Chu et al., 2008; Mariona, 2016; Robbins et al., 2014). However, the prevalence and implications of postpartum weight retention in low‐ and middle‐income countries, particularly in the context of the HIV epidemic, has received less attention (Ramlal et al., 2013; Widen et al., 2017). In our analysis, we estimated that 51% of women were obese by 12 months postpartum, including 43% of HIV‐infected women. In addition, similar to previous studies, both HIV‐uninfected and HIV‐infected women tended to gain, rather than lose weight, in the first year postpartum (Chetty et al., 2014; Murnane et al., 2010). For HIV‐uninfected women, postpartum weight gain translated into the median BMI moving from “overweight” (median BMI 28.7) at 6 weeks postpartum to “obese” (median BMI 31.6) at 12 months postpartum. For HIV‐infected women, the median BMI was overweight throughout the postpartum period. Weight at 12 months postpartum is a risk factor for obesity, which over times increases the risk of hypertension, Type 2 diabetes, cardiovascular disease later in life, and obesity in subsequent pregnancies (Catalano & Shankar, 2017; Endres et al., 2015; Kew et al., 2014; Puhkala, Luoto, Ahotupa, Raitanen, & Vasankari, 2013).

In settings such as South Africa where both HIV and obesity are prevalent, obesity during pregnancy and postpartum may exacerbate complications associated with HIV infection and ART use. In non‐pregnant adults with HIV, some types of ART, most notably some protease inhibitors, are well known to increase central adiposity, dyslipidemia, insulin resistance, and the subsequent risk of diabetes and cardiovascular disease (Beraldo et al., 2018; Kamin & Grinspoon, 2005). Thus, postpartum weight retention among HIV‐infected women may further exacerbate the detrimental cardiometabolic effects of HIV infection and ART use. For HIV‐infected pregnant women, ART has been linked to an increased risk of gestational diabetes (Gonzalez‐Tome et al., 2008; Jao et al., 2013; Marti et al., 2007; Soepnel et al., 2017) and postpartum weight gain in women with a BMI >25 (Cames et al., 2014). The effect of HIV infection and ART use on the development of hypertensive disorders during pregnancy, including preeclampsia, remains less clear (Adams, Watts, & Phelps, 2016; Browne, Schrier, Grobbee, Peters, & Klipstein‐Grobusch, 2015; Hall, Gebhardt, Theron, & Grove, 2014; Machado et al., 2014; Stoner et al., 2016). More recently, dolutegravir, an integrase inhibitor set to roll out in many low‐ and middle‐income countries, has been linked to increased weight gain in adults living with HIV, with the highest weight gain seem among women (Menard et al., 2017; NAMSAL ANRS 12313 Study Group et al., 2019; Norwood et al., 2017; Taramasso et al., 2017; Venter et al., 2019). Dolutegravir was recently recommended as first‐line therapy for pregnant and breastfeeding women living with HIV (The World Health Organization, 2019), raising further concerns about weight gain during pregnancy and postpartum.

HIV‐infected women initiating ART in our study were more likely to have Stage 1 or 2 hypertension at enrollment into ANC (33 vs. 22%) and to report hazardous alcohol use (25 vs. 7%) compared with HIV‐uninfected women. Alcohol use in our study was measured using a validated tool (Bush et al., 1998) but may be under‐reported. HIV infection, ART use, and high levels of alcohol consumption are all associated with hypertension (Briasoulis, Agarwal, & Messerli, 2012; Fahme, Bloomfield, & Peck, 2018) and may help to explain the higher levels of hypertension observed among HIV‐infected women in our cohort. Unfortunately, we did not have information on the development of gestational diabetes, pregnancy‐induced hypertension, or preeclampsia or postpartum hypertension or diabetes and could not examine possible downstream effects of hypertension at baseline or associations between pre‐pregnancy BMI category and the development of pregnancy complications.

In our analysis, being married or cohabitating, higher gravidity, and having high blood pressure at enrollment into ANC were associated with an increased risk of being obese II or III at 12 months postpartum, whereas being HIV infected was associated with a reduced risk of obesity at 12 months postpartum; HIV infection has been associated in previous studies in sub‐Saharan Africa with being underweight (Grinspoon et al., 1998; Karim et al., 2011; Kindra et al., 2011; Lartey, 2008; Villamor et al., 2006; Widen et al., 2013; Widen et al., 2019; Young et al., 2012). Here, only 1% of HIV‐infected women were underweight at their first ANC visit, whereas 43% were obese I‐III. Despite the high prevalence of obesity, HIV‐infected women were less likely than HIV‐uninfected women to be obese II or III at 12 months postpartum, which may, in part, reflect natural heterogeneity in postpartum weight retention. Unlike others in high‐resource settings, baseline education and employment status were not associated with obesity status at 12 months postpartum, highlighting the complexity of factors associated with obesity overtime (Gaillard et al., 2013).

Strengths of this analysis included the availability of detailed sociodemographic, clinical, behavioural, and HIV‐related characteristics that could be related to obesity in a large cohort of HIV‐infected and HIV‐uninfected women. In addition, the ability to compare directly measures of maternal anthropometry at enrollment into ANC and through 12 months postpartum between HIV‐infected and HIV‐uninfected women is unique. Limitations include the lack of information on obstetric morbidity such as gestational diabetes or hypertension, as well as measured pre‐pregnancy BMI and gestational weight gain during pregnancy. The median gestational age at enrollment into ANC in our cohort was 20 weeks, past the first trimester of pregnancy when ultrasound is the most accurate for estimating gestational age (Butt & Lim, 2014). However, recent evidence supports the accuracy of ultrasound to estimate gestational age after the first trimester during pregnancy (Butt & Lim, 2014; Papageorghiou et al., 2016). Finally, we note as a limitation that breastfeeding duration and modality was not accounted for in the analysis due it being an intermediary variable between several predictors examined and obesity at 12 months postpartum. In South Africa, nearly 60% of women exclusively breastfeed in early infancy (Jackson et al., 2019). However, in one study, HIV‐infected women in South Africa were less likely to exclusively breastfeed compared with HIV‐uninfected women; but duration of exclusive breastfeeding did not differ by HIV status (Chetty et al., 2014). Duration and modality of breastfeeding through 12 months was likely heterogeneous in this population and could have played an important role in the weight gain patterns.

## 5 CONCLUSION

Throughout sub‐Saharan Africa, the dual burden of infectious and chronic diseases remains a major focus of efforts to improve maternal and child health. In some areas of South Africa, obesity affects nearly half of all pregnant women, and postpartum weight retention is a growing concern. HIV infection and its treatment may contribute to weight gain and metabolic changes during pregnancy and postpartum that could further exacerbate the adverse effects of obesity on maternal and child health outcomes. For these reasons, the combined impact of obesity and HIV infection in South Africa is likely to have important implications for the health of women and their children during pregnancy and beyond. Our analysis beings to address these concerns by describing maternal anthropometry and factors associated with being overweight or obese at 12 months postpartum in a cohort of HIV‐infected and HIV‐uninfected women. Future studies of the prevalence and impact of obesity during pregnancy and postpartum weight retention in low‐resource settings with a high burden of HIV are urgently needed to generate an evidence‐base to guide clinical decision‐making, prevention efforts, and public health interventions to optimize maternal and child health outcomes in the coming years.

## CONFLICTS OF INTEREST

The authors declare that they have no conflicts of interest.

## CONTRIBUTIONS

TKP, SML, EJA, and LM designed the study and oversaw data collection. AMB analyzed the data and drafted the manuscript. All authors critically reviewed the manuscript.

## Supporting information


**Table S1.** Multivariable associations for the relationship between baseline characteristics with being overweight/obese I or obese II/III, compared to underweight or normal (referent) at 12 months postpartum after using multiple imputation to address missing data
**Table S2. B**ivariable and multivariable associations for the relationship between characteristics at enrollment into antenatal care and weight los) or weight gain, compared to no change in weight between 6 weeks postpartum and 12 months postpartum among 596 HIV‐infected and HIV‐uninfected women with weight measures at 6 weeks postpartum and 12 months postpartum.
**Table S3.** Bivariable associations for the relationship between baseline HIV characteristics with being overweight/obese I or obese II/III, compared to underweight or normal (referent) at 12 months postpartum among HIV‐infected women (N = 464)
**Table S4.** Maternal Anthropometry Information among women with complete BMI data (N = 458)
**Table S5.** Missing BMI data, among people who completed each visit
