# Supplementary material for: Postpartum obesity and weight gain among human immunodeficiency virus‐infected and human immunodeficiency virus‐uninfected women in South Africa
Source: Matern Child Nutr. 2020 Jan 13;16(3):e12949. doi: 10.1111/mcn.12949 (PMC7296802; doi:10.1111/mcn.12949)
Supplement: Supplementary file 1 — Table S1. Multivariable associations for the relationship between baseline characteristics with being overweight/obese I or obese II/III, compared to underweight or normal (referent) at 12 months postpartum after using multiple imputation to address missing data Table S2. Bivariable and multivariable associations for the relationship between characteristics at enrollment into antenatal care and weight los) or weight gain, compared to no change in weight between 6 weeks postpartum and 12 months postpartum among 596 HIV‐infected and HIV‐uninfected women with weight measures at 6 weeks postpartum and 12 months postpartum. Table S3. Bivariable associations for the relationship between baseline HIV characteristics with being overweight/obese I or obese II/III, compared to underweight or normal (referent) at 12 months postpartum among HIV‐infected women (N = 464) Table S4. Maternal Anthropometry Information among women with complete BMI data (N = 458) Table S5. Missing BMI data, among people who completed each visit [file MCN-16-e12949-s001.docx]

**Appendix: Supplemental Tables**

| **Table S1.** Multivariable associations for the relationship between baseline characteristics with being overweight/obese I or obese II/III, compared to underweight or normal (referent) at 12 months postpartum after using multiple imputation to address missing data | | |
| --- | --- | --- |
|  | **Overweight or obese I (BMI 25 - <35)** | **Obese II or III  (BMI > 35)** |
| **Baseline Characteristics** | **OR (95% CI)** | **OR  (95% CI)** |
| HIV-status |  |  |
| HIV-uninfected | 1.00 | 1.00 |
| HIV-infected | 0.74 (0.49, 1.13) | **0.41 (0.25, 0.70)** |
| Employment |  |  |
| Unemployed | 1.00 | 1.00 |
| Employed | 1.45 (0.98, 2.15) | 1.36 (0.86, 2.15) |
| Marital status |  |  |
| Not married/cohabitating | 1.00 | 1.00 |
| Married/cohabitating | 1.40 (0.94, 2.09) | **2.38 (1.50, 3.78)** |
| Gravidity, continuous | **1.24 (1.04, 1.50)** | **1.30 (1.06, 1.60)** |
| Blood pressure category |  |  |
| Normal | 1.00 | 1.00 |
| Elevated | 1.68 (0.99, 2.83) | **3.01 (1.68, 5.39)** |
| Stage 1 or 2 high | **2.36 (1.37, 4.05)** | **5.62 (3.09, 10.23)** |
| OR = odds ratio; BMI = body mass index. Bold indicates p-value <0.05. Multivariable associations are adjusted for all other covariates listed in the table. N=50 imputations. | | |

| **Table S2. B**ivariable and multivariable associations for the relationship between characteristics at enrollment into antenatal care and weight los) or weight gain, compared to no change in weight between 6 weeks postpartum and 12 months postpartum among 596 HIV-infected and HIV-uninfected women with weight measures at 6 weeks postpartum and 12 months postpartum. | | | | | | |
| --- | --- | --- | --- | --- | --- | --- |
|  | **Weight loss** | |  | **Weight gain** | |  |
| **Characteristics at enrollment  into antenatal care** | **Bivariable OR  (95% CI)** | **Multivariable OR (95% CI)** |  | **Bivariable OR  (95% CI)** | **Multivariable OR  (95% CI)** | |
| HIV-status |  |  |  |  |  | |
| HIV-uninfected | 1.00 | -- |  | 1.00 | -- | |
| HIV-infected | 1.30 (0.81, 2.09) | -- |  | 0.82 (0.55, 1.22) | -- | |
| Education |  |  |  |  |  | |
| Less than secondary | 1.00 | -- |  | 1.00 | -- | |
| Secondary or higher | 0.98 (0.59, 1.61) | -- |  | 1.05 (0.69, 1.60) | -- | |
| Employment |  |  |  |  |  | |
| Unemployed | 1.00 | -- |  | 1.00 | -- | |
| Employed | 1.22 (0.75, 1.98) | -- |  | 1.41 (0.93, 2.11) | -- | |
| Poverty category |  |  |  |  |  | |
| Most disadvantaged | 1.00 | -- |  | 1.00 | -- | |
| Moderate disadvantage | 0.89 (0.50, 1.58) | -- |  | 0.72 (0.45, 1.14) | -- | |
| Least disadvantaged | 1.65 (0.88, 3.09) | -- |  | 1.10 ( 0.64, 1.87) | -- | |
| Planned current pregnancy |  |  |  |  |  | |
| No | 1.00 | -- |  | 1.00 | -- | |
| Yes | 0.98 (0.60, 1.61) | -- |  | 0.86 (0.57, 1.31) | -- | |
| Marital status |  |  |  |  |  | |
| Not married/cohabitating | 1.00 | -- |  | 1.00 | -- | |
| Married/cohabitating | 0.82 (0.51, 1.34) | -- |  | 1.24 (0.83, 1.86) | -- | |
| Maternal age | 0.99 (0.95, 1.03) | -- |  | 0.99 (0.96, 1.03) | -- | |
| Gravidity, continuous | 0.95 (0.78, 1.15) | -- |  | 1.06 (0.91, 1.25) | -- | |
| Primigravida |  |  |  |  |  | |
| No | 1.00 | 1.00 |  | 1.00 | 1.00 | |
| Yes | 2.16 (1.18, 3.97)* | 2.69 (1.38, 5.25)* |  | 1.48 (0.85, 2.55) | 1.79 (0.98, 3.28) | |
| Perinatal depression |  |  |  |  |  | |
| No probable depression | 1.00 | -- |  | 1.00 | -- | |
| Probable depression | 1.33 (0.60, 2.97) | -- |  | 0.74 (0.35, 1.55) | -- | |
| AUDIT-C categories |  |  |  |  |  | |
| Below threshold | 1.00 | 1.00 |  | 1.00 | 1.00 | |
| Hazardous drinking | 2.80 (1.42, 5.53)* | 3.22 (1.53, 6.77)* |  | 1.64 (0.87, 3.08) | 1.51 (0.76, 3.00) | |
| Blood pressure category |  |  |  |  |  | |
| Normal | 1.00 | 1.00 |  | 1.00 | 1.00 | |
| Elevated | 1.22 (0.64, 2.31) | 1.19 (0.62, 2.29) |  | 1.39 (0.70, 2.44) | 1.35 (0.76, 2.38) | |
| Stage 1 or 2 high | 0.67 (0.34, 1.31) | 0.61 (0.30, 1.23) |  | 2.00 (1.19, 3.34)* | 1.97 (1.17, 3.31)* | |
| BMI = body mass index; OR = odds ratio. Weight change between 6 weeks postpartum and 12 months postpartum were defined as no weight change (within +/- 2 kilograms (kgs) of weight at 6 weeks postpartum), weight loss more than 2 kgs, or weight gain more than 2kgs. Multivariable associations are adjusted for all other covariates listed in the table. * indicates p-value <0.05 | | | | | | |

| **Table S3.** Bivariable associations for the relationship between baseline HIV characteristics with being overweight/obese I or obese II/III, compared to underweight or normal (referent) at 12 months postpartum among HIV-infected women (N=464) | | |
| --- | --- | --- |
|  | **Overweight or obese I (BMI 25 - <35)** | **Obese II or III  (BMI > 35)** |
| **Baseline Characteristics** | **OR (95% CI)** | **OR  (95% CI)** |
| HIV diagnosis |  |  |
| Before this pregnancy | 1.00 | 1.00 |
| During this pregnancy | 0.93 (0.57, 1.53) | 0.71 (0.39, 1.30) |
| PMTCT prophylaxis in a previous pregnancy |  |  |
| No | 1.00 | 1.00 |
| Yes | 1.14 (0.63, 2.06) | 1.55 (0.77, 3.11) |
| Viral load, copies/ml |  |  |
| <1,000 | 1.00 | 1.00 |
| 1,000 - <10,000 | 1.29 (0.57, 2.90) | 0.87 (0.35, 2.15) |
| > 10,000 | 1.01 (0.48, 2.17) | 0.52 (0.22, 1.22) |
| CD4 count, cells/mm^3^ |  |  |
| <200 | 1.00 | 1.00 |
| 201 - <350 | 1.21 (0.58, 2.55) | 1.52 (0.59, 3.92) |
| 351- <500 | 0.81 (0.36, 1.79) | 1.20 (0.44, 3.27) |
| >500 | 0.72 (0.33, 1.54) | 0.93 (0.35, 2.48) |
| OR = odds ratio; ; BMI = body mass index; PMTCT = prevention of mother-to-child HIV transmission. | | |

**Table S4. Maternal Anthropometry Information among women with complete BMI data (N=458)**

| **A.** | **Maternal weight (kg) - Median (IQR)** | | |
| --- | --- | --- | --- |
|  | 6 weeks postpartum | 6 months postpartum | 12 months postpartum |
| HIV-uninfected | 76.1 (66.1, 90.0) | 78.3 (66.7, 94.0) | 78.8 (67.0, 96.0) |
| HIV-infected | 72.0 (62.1, 81.7) | 71.9 (62.0, 83.6) | 72.4 (62.0, 87.0) |
| **B.** | **Maternal BMI^1^ - Median (IQR)** | | |
|  | 6 weeks postpartum | 6 months postpartum | 12 months postpartum |
| HIV-uninfected | 29.9 (26.1, 35.3) | 31.2 (26.8, 36.9) | 31.6 (27.0, 37.3) |
| HIV-infected | 28.1 (24.8, 32.4) | 28.7 (24.1, 33.0) | 28.8 (24.4, 35.2) |
| **C.** | **Maternal weight change^2^, from 6 weeks postpartum (kg) - Median (IQR)** | | |
|  | 6 months postpartum | 9 months postpartum | 12 months postpartum |
| HIV-uninfected | 2.1 (-0.8, 5.5) | 3.1 (-1.0, 7.2) | 3.5 (-0.9, 8.2) |
| HIV-infected | 0.05 (-2.0, 3.3) | 0.8 (-2.4, 5.9) | 1.3 (-2.2, 7.0) |
| ^1^BMI categories are: underweight (<18.5), normal (18.5 - <25), overweight (25 - <30), obese I (30 - <35), obese II (35 - <40), obese III (>40). ^2^Negative value indicates weight loss from 6 weeks postpartum; positive value indicates weight gain from 6 weeks postpartum. | | | |

| **Table S5. Missing BMI data, among people who completed each visit** | | |  |  |  |  |  |  |  |
| --- | --- | --- | --- | --- | --- | --- | --- | --- | --- |
|  | Enrollment | | | 6 weeks pp | | | 3 mo pp | | |
|  | N | Total | % | N | Total | % | N | Total | % |
| HIV-uninfected | 14 | 413 | 3.4 | 10 | 391 | 2.6 | 6 | 370 | 1.6 |
| HIV-infected | 74 | 464 | 15.9 | 109 | 439 | 24.8 | 16 | 370 | 4.3 |
|  |  |  |  |  |  |  |  |  |  |
|  | 6 mo pp | | | 9 mo pp | | | 12 mo pp | | |
|  | N | Total | % | N | Total | % | N | Total | % |
| HIV-uninfected | 8 | 348 | 2.3 | 4 | 332 | 1.2 | 4 | 355 | 1.1 |
| HIV-infected | 32 | 404 | 7.9 | 37 | 386 | 9.6 | 47 | 388 | 12.1 |
